# Measuring the Impact of Future Outbreaks? A Secondary Analysis of Routinely Available Data in Spain

**DOI:** 10.3390/ijerph192113981

**Published:** 2022-10-27

**Authors:** Jimmy Martin-Delgado, Aurora Mula, Rafael Manzanera, Jose Joaquin Mira

**Affiliations:** 1 Hospital Luis Vernaza, Junta de Beneficencia de Guayaquil, Guayaquil 090306, Ecuador; 2 Instituto de Investigación e Innovación en Salud Integral, Facultad de Ciencias Médicas, Universidad Católica de Santiago de Guayaquil, Guayaquil 090603, Ecuador; 3 Atenea Research Group, Foundation for the Promotion of Health and Biomedical Research, 03550 Sant Joan d’Alacant, Spain; 4 Health and Economics Benefits Area, MC Mutual, 08037 Barcelona, Spain; 5 Health Psychology Department, Miguel Hernández University, 03202 Elche, Spain; 6 Health District Alicante-Sant Joan, 03013 Alicante, Spain

**Keywords:** health services, COVID-19, health management, epidemiologic factors, socioeconomic factors

## Abstract

**Background:** As of 7 January 2022, it is estimated that 5.5 million people worldwide have died from COVID-19. Although the full impact of SARS-CoV-2 (COVID-19) on healthcare systems worldwide is still unknown, we must consider the socio-economic impact. For instance, it has resulted in an 11% decrease in the GDP (Gross Domestic Product) in the European Union. We aim to provide valuable information for policymakers by analysing widely available epidemiological and socioeconomic indicators using Spanish data. **Methods:** Secondary analysis of routinely available data from various official data sources covering the period from 1 March 2020 to 31 March 2021. To measure the impact of COVID-19 in the population, a set of epidemiological and socioeconomic indicators were used. The interrelationships between these socioeconomic and epidemiological indicators were analysed using Pearson’s correlation. Their behaviour was grouped according to their greater capacity to measure the impact of the pandemic and was compared to identify those that are more appropriate to monitor future health crises (primary outcome) using multivariate analysis of canonical correlation for estimating the correlation between indicators using different units of analysis. **Results:** Data from different time points were analysed. The excess of mortality was negatively correlated with the number of new companies created during the pandemic. The increase in COVID-19 cases was associated with the rise of unemployed workers. Neither GDP nor per capita debt was related to any epidemiological indicators considered in the annual analysis. The canonical models of socioeconomic and epidemiological indicators of each of the time periods analysed were statistically significant (0.80–0.91 *p* < 0.05). **Conclusions:** In conclusion, during the COVID-19 pandemic in Spain, excess mortality, incidence, lethality, and unemployment constituted the best group of indicators to measure the impact of the pandemic. These indicators, widely available, could provide valuable information to policymakers and higher management in future outbreaks.

## 1. Introduction

The full impact of SARS-CoV-2 (COVID-19) on healthcare systems worldwide is still unknown. We do know the number of people affected worldwide by COVID-19 and have estimates of the number of patients with other conditions and pathologies whose treatment was interrupted due to closures of facilities, cancellations or interventions and visits, or because patients avoided visiting healthcare facilities because of fear of contagion [1]. As of 7 January 2022, it is estimated that 5.5 million people worldwide have died from COVID-19 and that the number of infections exceeds 315 million cases. The United States, India and Brazil had the highest number of infections registered as of that date [2]. Spain was among the hardest-hit countries during the first wave of COVID-19 [3]. As of 7 January 2022, 6.92 million cases and 89,837 deaths had been reported. This places Spain among the top five European countries by the number of cases [2].

Countries have adopted non-pharmaceutical interventions (NPIs) to contain the pandemic [4]. These NPIs consist of measures such as mandatory use of masks in enclosed places; physical distance; closure of leisure and educational centres; teleworking; border restrictions; and, in the most extreme circumstances, lockdowns [5]. The implementation of NPIs has changed as the epidemiological situation of the pandemic has progressed due to the favourable response to the vaccination strategies [6]. In the first waves of the pandemic, hospital pressure determined the type and timing of implementing these measures to prevent transmission. Nevertheless, as vaccination has progressed, the measures adopted have also considered the impact on the economy. Maintaining non-pharmacological intervention is the best way to achieve protection, and flexibility in their implementation can yield containment with minimal harm to societies, economies, and mental health [7].

Many health systems have experienced major problems, being on the verge of collapse. This meant a deterioration in the quality of care, the impossibility of attending to all patients (scarcity dilemma) and the neglect of patients with pathologies other than COVID-19, which will put greater pressure on health systems in the future [8,9]. Different studies have analysed the relationship between health expenditure and mortality from COVID-19. In a study by Elola-Somoza, no relationship was found between ‘low’ health expenditure and mortality from COVID-19 [10]. In another study that included 40 countries, no differences were found between the capacity to respond to the pandemic and broad adoption of COVID-19 testing and the adoption of other early, strict public health measures [11]. Another study conducted in Spain found that the number of physicians per 100,000 inhabitants, the proportion of nursing homes >100 beds, and urban population as significant at different levels for mortality rate [12]. When addressing COVID-19 transparency of health management matters. As the pandemic progressed in Spain there is a deterioration in citizen satisfaction with the healthcare management and the services provided, as well as in the well-being generated by them [13].

However, beyond the NPIs implemented and the evolution of the epidemiological situation, there is also the socioeconomic impact of COVID-19 on the world. In the European Union, it has resulted in an increase in inflation of 2.4% compared to March 2020, an 11% decrease in the GDP (Gross Domestic Product) in the second quarter of 2020 and economic efforts in all sectors (social security, health, companies and reactivation) to alleviate the impact of the pandemic, estimated at 3.7 trillion euros [14,15]. This impact is similar and sometimes more profound in other areas: a 6.3% decrease in the economy in Latin America and the Caribbean, a 3.4% decrease in the GDP in the United States during 2020 [16], and an overall global economic decline of 17.2% in September 2020 [17].

A broad set of indicators have been used to decide how to face the outbreak impact. Even though other efforts have been made this has been made with cross sectional data. It’s important to know which combined set of epidemiological and socio-economic indicators best reflects the scope and severity of the impact of the pandemic. Furthermore, the most significant indicators have changed as the virus and its transmission, severity, and lethality have evolved.

This study describes the socioeconomic and epidemiological impacts of the COVID-19 pandemic in Spain up to the first quarter of 2021 by means of traditionally used indicators. As a secondary objective, from the analysis of these data, the relationships between the indicators used to measure the impact of the pandemic were analyzed. In addition, the groupings that allow monitoring the impact of the COVID-19 pandemic over time were identified. We aim to provide valuable information for policymakers worldwide by means of given interpretative data using widely available indicators.

## 2. Materials and Methods

A secondary analysis of the data was conducted to measure the impact of the COVID-19 pandemic in Spain. Information was obtained from all the 17 autonomous communities of Spain and the autonomous cities of Ceuta and Melilla, which were considered as the units of analysis. The data was extracted from multiple official data sources allowed us, firstly, to calculate a set of epidemiological and socioeconomic indicators commonly used to establish the impact of the COVID-19 pandemic. Data extraction was done using the same definitions, criteria, and methodology across the data sources.

### 2.1. Time Periods Analysed

The calendar quarters of 2020 (Q1: January, February, March; Q2: April, May, June; Q3: July, August, September; Q4: October, November, December) and the first quarter of 2021 (Q1: January, February, March) were considered. The data were included in six datasets: annual data for 2020 and five quarterly data sets (Q1 2020, Q2 2020, Q3 2020, Q4 2020 and Q1 2021). The epidemiological data referring to COVID-19 for the first quarter of 2020 were collected from the beginning of the pandemic in Spain, adopting the first case’s initial date reported in RENAVE (*Red Nacional de Vigilancia Epidemiológica*) on 18 January 2020.

### 2.2. Setting

This study focused on the Spanish case. From 14 March 2020 to 2 May 2020, a period of strict lockdown was decreed to reduce the transmission rate, protect the population, and ensure that the health care system could provide care for the most significant number of COVID-19 cases. As of this date, the different units of analysis had the autonomy to establish measures to prevent infections.

Spain is constituted of 17 autonomous communities and two cities with autonomous status. It is essential to understand the organisation of the Spanish territory, given that each autonomous community in Spain can independently direct their social, economic and health organisations, except for Ceuta and Melilla, where the Ministry of Health manages the health system. Among these attributions, we find the NPIs to contain COVID-19. Since May 2020, each of the units of analysis has had the power to issue NPIs that respond to the specific situation in their territories. See Supplementary Material Table S1 for the different measures taken.

### 2.3. Data Sources

#### 2.3.1. Structure of the Health System

The number of hospital beds, the number of primary care professionals and the number of hospital care professionals were measured pre-COVID-19 as baseline data. The databases of the Ministry of Health [13] were used to obtain the data (the year 2019): ratio of primary care professionals per 100,000 inhabitants, rate of beds per 1000 inhabitants, and the ratio of professionals (medical doctors and nurses) in hospitals per bed (see Table 1).

#### 2.3.2. Epidemiological Data

All data were obtained from the National Epidemiology Centre and the Carlos III Health Institute, based on the number of COVID-19 cases reported in RENAVE [18], which periodically updates the COVID-19 situation in Spain. We included: number of COVID-19 cases, number of patients hospitalised for COVID-19, number of patients admitted to Intensive Care Units (ICUs) for COVID-19, and number of patients who died from COVID-19. Patients on the waiting list were obtained from the Ministry of Health between June 2019 and June 2020. Expected and observed deaths were obtained from the Daily Mortality Monitoring System (*Monitorización de Mortalidad diaria*) of the Carlos III Health Institute [19].

#### 2.3.3. Socioeconomic Data

The number of inhabitants, the territorial area and the ageing index as of 01/01/2020 was obtained from the National Institute of Statistics (*Instituto Nacional de Estadística*—INE) [20]. The number of unemployed people was obtained from INE. The number of people in ‘Temporary Employment Regulation’ (*Expediente de Regulación Temporal de Empleo*—ERTE) is an instrument used by businesses in which Social Security would take up payroll payments of employees over a period of time. Although, in practice, these workers in ERTE are assimilated to unemployment were taken from the General Treasury of the Social Security. Data on companies registered with Social Security were obtained from the Ministry of Labour and Social Economy database [21]. In contrast, data on new companies created were obtained from the databases of the INE. Data on debt-to-GDP ratio and debt per capita were extracted from Available online: https://datosmacro.expansion.com (accessed on: 5 July 2021).

### 2.4. Construction of Indicators

We constructed indicators based on the formulas described in Table 1. All the indicators were calculated quarterly and annually for the 2020 period and each autonomous community. The datasets are available in an open repository [22].

*Epidemiological indicators:* the following COVID-19 indicators were included, incidence, severity index, hospitalisation rate, bed saturation, ICU bed saturation, lethality, mortality, rate of ICU admissions and cases. Other indicators were excess mortality, excess of patients on the waiting list, and non-COVID-19 excess mortality.

*Socioeconomic indicators:* population density, unemployment rate, number of people in the ERTE, percentage of variation in the number of companies registered with the Social Security, number of companies that closed for any reason, and percentage of variation in the creation of new companies (Table 1).

### 2.5. Statistical Analysis

The relationship between the available resources (structural variables) and epidemiological indicators were determined using the Pearson’s coefficient (correlation matrix of the 2020 annual dataset). The relationship between the socioeconomic and epidemiological indicators was analysed using Pearson’s correlation matrices of both sets of indicators for each dataset.

A multivariate analysis of canonical correlation analysis was conducted to find the linear combination of values that maximised the correlation between both sets of indicators while allowing for different units of analysis across indicators. Canonical correlations for each dataset (Q1, Q2, Q3, Q4, Annual 2020 and Q1 2021) were calculated, with canonical loads reflecting the variance that the observed variable shared with the canonical theoretical value and their magnitude corresponding to their predictive capacity for a given set of indicators. The indicators included were incidence, saturation of hospital beds, lethality, COVID-19 cases admitted to the ICU, excess mortality, unemployment rate, variation of companies registered with the Social Security and variation of new companies created, typified by their mean and divided by their standard deviation. Excess patients on the waiting list, GDP, and per capita debt were not included because data was only available for a whole annual. Due to multicollinearity, the hospitalisation rate, mortality, severity index, and ICU bed saturation indicators were excluded from the analysis.

The statistical analyses were carried out in the RStudio programming environment, and the CCA [23] package was used to calculate the canonical correlations. Statistical significance was set at *p* < 0.05.

## 3. Results

At the beginning of the pandemic, the rate of beds per 1000 inhabitants in Spain was 2.4. The ratio of primary care professionals per 100,000 inhabitants was 171.8. The ratio of hospital professionals per bed was 2.1. As of 31 March 2021 (end of Q1 2021), the number of people diagnosed with COVID-19 in Spain totalled 3,278,056. On the last day of the analysed period, the number of deaths officially attributed to COVID-19 totalled 76,293. The incidence of cases between the Q1 and Q2-2020 periods remained stable, even though mortality doubled. During Q2-2020, the highest excess mortality was observed (24,236 deaths). The Q3-2020 period had the lowest mortality among the periods analysed (183 per 100,000 inhabitants). In Q1-2021, the incidence of cases and mortality was maintained compared to Q4-2020 (Supplementary File, Tables S2 and S3).

The unemployment rate in Spain stood at 14.1%, and the per capita debt at the end of 2019 was 25,116 euros. The unemployment rate as of 31 March 2021 had increased by 1.6% concerning the data before the start of the pandemic. The number of people in the ERTE in December 2020 totalled 702,808 workers. In the same period, the highest average number of workers in the ERTE was observed at 2.5 million people. This is 46 times more workers compared to the last data registered in December 2019, before the pandemic. The number of workers in the ERTE fluctuated between the periods, starting in Q1 with 3.1 million people and dropping to 674,366 people by the end of the study period, in Q1-2021.

The most significant reduction in the number of companies was observed in Q2-2020 (−6.02%). Even though the number of companies has grown since Q2-2020, the highest percentage of unemployment was observed in Q1-2021 (Supplementary File, Table S4). The number of companies decreased by 1.2%, and the per capita debt at the end of 2020 had increased by 3272 euros compared to a year earlier.

### 3.1. Relationships between the Epidemiological Indicators

As expected, the hospitalisation rate was correlated with the overall saturation of beds and the saturation of ICU beds in the 2020 annual dataset (r = 0.92 and r = 0.80, respectively). This pattern was replicated across all five time periods considered (Supplementary Material). The excess mortality was associated with COVID-19 incidence and lethality, a pattern observed in three of the five periods. The severity index was related to lethality (r = 0.73) and saturation of ICU beds with incidence (r = 0.78) also in three of the five periods. However, in the first quarter of 2021, the saturation of ICU beds was not related to the incidence (Supplementary Material, Tables S5 and S6).

### 3.2. Relationships between the Socioeconomic Indicators

A high correlation between the GDP and DPC (Debt per Capita) and between unemployment and the number of companies was observed in the 2020 annual dataset. This last pattern was observed in four of the five quarterly periods (Supplementary Material, Figures S1–S6).

### 3.3. Relationship between Health System Structure and Epidemiological Indicators

The aging index was correlated (r = 0.55, *p* < 0.05) with the severity index and with lethality (r = 0.67, *p* < 0.05) considering the 2020 data. Furthermore, the number of beds was related to lethality (r = 0.52, *p* < 0.05). Finally, there was a higher ratio of professionals in primary care with a higher lethality (r = 0.51, *p* < 0.05). Greater availability of healthcare structure resources (number of beds and professionals) before the first wave is not associated with less mortality.

### 3.4. Relationship between Epidemiological and Socioeconomic Indicators

As a result of COVID-19, mortality or hospitalisations increased, unemployment increased, and the number of new businesses decreased. The severity of infection and lethality were higher in the units of analysis with more ageing (older population). The number of companies in the Social Security registry and the reduction in unemployment grew in parallel with the hospitalisation and mortality rates. The increase in the number of COVID-19 cases was correlated with the increase of workers in the ERTE. Neither GDP nor per capita debt was related to any epidemiological indicators considered in the annual analysis (Table 2).

In 2021, it was observed that when the saturation of ICUs increased, the number of companies created tended to decrease; that the number of diagnosed cases of COVID-19 grew in parallel with the number of workers in the ERTE; and that excess mortality from causes not attributed to COVID-19 and the number of people in the ERTE were positively correlated. In the different periods of 2020, the correlation between the ERTE and COVID-19 cases was constant. However, in each analysis period, differences were observed in the interrelationships between the indicators (Supplementary File includes Tables S5 and S6 and all correlations of the socioeconomic and epidemiological indicators for each of the periods considered in this analysis).

### 3.5. Relationship between Groups of Epidemiological and Socioeconomic Indicators (Canonical Correlations)

**Figure 1:**
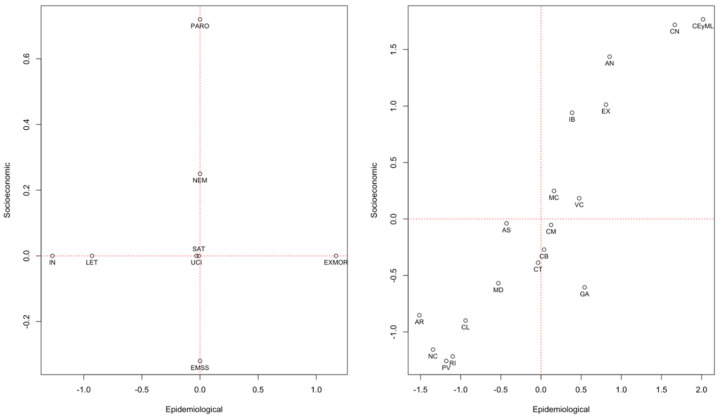
Graphical representation of the canonical correlations of the socioeconomic and epidemiological indicators during 2020 annual data. AN: Andalusia; AR: Aragon; AS: Asturias; IB: Balearic Islands; CN: Canary Islands; CB: Cantabria; CM: Castilla La-Mancha; CL: Castilla y León; CT: Catalonia; CEyML: Ceuta and Melilla; VC: Community of Valencia; EX: Extremadura; GA: Galicia; RI: La Rioja; MD: Community of Madrid; MC: Murcia; NC: Navarra; PV: Basque Country. Socioeconomic Indicators (PARO: unemployment rate; EMSS: percentage change in companies registered with Social Security; NEM: percentage change in new companies created). Epidemiological Indicators (IN: incidence; SAT: bed saturation; LET: lethality; ICU: rate of COVID-19 admissions among COVID-19 cases; EXMOR: excess mortality).

The first model with the set of 2020 annual data was statistically significant (0.91, *p* < 0.05), as were the following equivalent models for: Q1-2020 (0.82, *p* < 0.05), Q2-2020 (0.83, *p* < 0.05), Q3-2020 (0.87, *p* < 0.05), Q4-2020 (0.80, *p* < 0.05) and Q1-2021 (0.83, *p* < 0.05). Table 3 shows the canonical loads of each model analysed according to the study period and the indicators included. The graphic representation of each model is available in the Supplementary Material. In the left graph of Figure 1, the model locates the epidemiological and socioeconomic indicators considering their weights in that period for each of the units of analysis. The graph on the right positions each autonomous community on the plane using a relationship directly proportional to the indicators of greater weight for that community.

In 2020, the resulting analysis units grouped the units of analysis into three groups according to the impact measures of the COVID-19 pandemic. In the cities of Ceuta and Melilla, the impact was fundamentally characterised by higher excess mortality and unemployment. In Murcia and Valencia, it was due to a higher saturation of ICUs and a lower variation in the number of registered companies, while in Castilla La Mancha, Cantabria, and Catalonia, it was due to the saturation of ICUs and lower unemployment. Lastly, the impact on the Basque Country, Navarra and Rioja was characterised by a higher incidence and lower lethality, while the number of companies was maintained (Figure 1). Supplementary Material shows the results of comparing the analysis units’ behaviour in the set of epidemiological and socioeconomic indicators considered using the graphical representation of the canonical correlations of the socioeconomic and epidemiological indicators.

## 4. Discussion

The consequences of the infection caused by the new variant of coronavirus have been harrowing in Spain. As of 7 January 2022, a total of 89,837 people had officially died from COVID-19, and the number of diagnosed cases reached 6.92 million. In socio-economic terms, debt in relation to the GDP has reached 122.80%, representing an increase of 27.3%. The number of companies registered with Social Security has decreased by 4.87% compared to 2019. Other studies have shown that countries in Oceania and Asian outperformed countries in other regions for pandemic containment prior to vaccine development [7,11].

This study was carried out after successive waves of the pandemic to harness the available information. It has a focus on measures related to agility in government response to implement non-pharmacological measures [11], social vulnerability [24] and combines several epidemiological and socioeconomic indicators in line with what has been suggested by other studies [3,10,25] but with a greater number of variables in the algorithms. We have included information from different time points in order to provide a better understanding of the socioeconomic variables around the pandemic. The Spanish case is particularly useful for this type of analysis because reliable primary data is updated periodically through proven procedures. The country has a political structure that has delegated health decisions to the autonomous governments. Therefore, the impact of the pandemic can be compared by considering different infection prevention measures (either by their approach or by the intensity of their application) throughout the harshest period of the pandemic.

The lethality in the study period was 2.33% and appeared to be consistently associated with the ageing of the population along all the timepoints analysed, reflecting how the impact of the pandemic has been particularly devastating among older people. We have found especially useful the aging index. This average indicates the proportion of people over 64 years of age. It was included in the analyses because it indicates in which Autonomous Community there were more people at risk of suffering serious consequences of COVID as a ratio of the total population. During the first and second quarters of 2020, a lower incidence of new cases was observed than in subsequent periods, which may be explained by the lower diagnostic capacity during that period due to deficiencies in the availability of diagnostic tests. Still, there was a higher excess mortality compared to the other periods [26]. The elderly living in care homes were the most affected in this period. From an epidemiological point of view, the third quarter had the most negligible impact, probably due to the collaboration of the citizens that had just come out of a period of uncertainty and the constant bombardment of news about the effects of the pandemic [27]. In the fourth quarter of 2020, this situation changed, and the number of COVID-19 cases increased remarkably. However, the impact in socioeconomic terms was milder than at the beginning of the health emergency. In these two quarters of 2020, a higher incidence was identified among young people [28]. The number of hospitalisations was shown to be related to lethality but not to mortality. Greater availability of beds was associated with higher lethality, probably because these centres treated more complex cases fundamentally at the beginning of the pandemic. Other authors have found that higher densities of licensed physicians and of general practitioners are associated with lower excess mortality [25]. In our study there is a similar relationship between the ratio of professionals in hospitals and excess mortality, although it is not significant.

The impact of the pandemic caused an increase of 33.3% in the unemployment rate (when adding up the number of people officially in unemployment with those who joined the lists of the ERTE). It generated an increase of 11.5% in per capita debt (without considering the debt for the funds provided by the European Commission to cover excess spending in the pandemic period). The increase in debt relative to the GDP was not related to any epidemiological indicators. This remains controversial, as some authors have found similar results [10,25] and others have found differences between deprived and more wealthy areas [29]. However, a systematic review that included domains and variables of social vulnerability affecting COVID-19 such as population aged 65 years or older, unemployment rate, population living below poverty line and public health infrastructures found that higher social vulnerability experienced greater mortality rates during the COVID-19 pandemic [24]. The growth of per capita debt and the number of cases followed a similar trend, although this relationship was not significant. On the other hand, the number of workers in the ERTE increased in parallel with the growth of the incidence at the beginning of the pandemic. As has been previously mentioned, the number of businesses decreased by 4.87% compared to 2019, even though of the measures adopted to protect the economy. Excess mortality was also demonstrated to be an adequate indicator, given that any increase in this indicator was accompanied by a reduction in the number of new companies being created. In 2020, a higher saturation of ICUs was correlated with a lower speed in the creation of companies and with a more significant number of workers in the ERTE.

Determining the impact of the COVID-19 pandemic on health systems and ultimately in socio-economic terms will take more time. In this work, we have been able to identify indicators that are more sensitive to the changes that occur on a day-to-day basis during the pandemic. However, it should be considered that certain aspects are subject to a time lag that we will only be able to observe as the years go by [30]. Other authors have identified that the effect of non-pharmacological interventions (e.g., lockdowns) are only relevant for a couple of weeks, but that a synergistic response of different measures and interventions and a robust health system are crucial for the reduction of deaths [31].

The COVID-19 pandemic is a complex phenomenon and cannot be reduced only to the death rate. In another study, cultural dimensions were related to COVID-19 measures only when socio-economic indicators were not considered but lost their significance when socio-economic variables were entered into the models [32]. As in our study, other authors suggest more outcome variables such as excess mortality as well as the impact on the economy and citizens’ freedom should be considered [33,34,35].

Canonical correlations allow the analysis of multidimensional relationships between multiple independent variables and multiple dependent variables. In this way, it is possible to determine whether two sets of indicators have a relationship with each other and, if so, to determine the magnitude. The results of this analysis suggested that the combination of epidemiological and socioeconomic indicators that best describe the impact of the COVID-19 pandemic should include the incidence of cases, COVID-19 lethality, excess mortality, and unemployment.

Bed saturation, excess mortality and unemployment were the indicators that best represented the impact measure in the first period of the pandemic. Gradually as data from other periods were analysed, unemployment, incidence and lethality began to add a more significant explanatory burden within the model. Excess mortality proved to be an essential indicator of the model over time. If we consider the unemployment rate, case incidence, and excess mortality as key indicators of the impact of the pandemic, the results of this study suggest that in 2020, the analysis units that performed “better” were Basque Country, La Rioja, Navarra, Aragon, Castilla y León and Madrid. In the 2021 quarter analysed, Cantabria, the Basque Country, Madrid, and Navarra. When we analysed these same indicators jointly throughout 2020, the Basque Country, Madrid, La Rioja and Navarra were the units of analysis with the “best” results. This coincides with the communities previously identified that first pushed Rt below 1, for example the Community of Madrid [36].

These indicators, which may be available in most countries, can be helpful in decision-making. Undoubtedly, other variables such as diagnostic capacity [37], communication [38], the effectiveness of the political leadership in each territory [39] and vaccination strategies [40,41] should be incorporated into the analysis. Incorporating these indicators into the decision-making process in future pandemics could be relevant in measuring the impact and adjusting the measures to contain the infection in different territories.

In the first wave, the availability of COVID-19 diagnostic tests conditioned the numbers of new COVID-19 cases, which affected the reliability of the incidence and mortality estimates. However, due to the significant discrepancy in the number of detection tests carried out among the units of analysis, these data have not been considered at different times during the pandemic. It has been pointed out in other studies that in Spain there has been a substantial delay between exposure and notification which could have impacted of the data assigned to each period. [36] The workers registered in the ERTE should be added to the unemployed to compare countries that do not have this social protection system. It must be noted that the primary data regarding the number of registered companies does not imply that they are operational since they can remain in the registry even if there is no economic activity. Given that resources were expanded, especially from Q2-2020, both due to the hiring of personnel and an increase in the number of beds, particularly in ICUs, the repetition of this analysis for other time periods was ruled out.

## 5. Conclusions

In conclusion, during the COVID-19 pandemic in Spain, excess mortality, case incidence, lethality and unemployment constituted the best group of indicators to measure the impact of the pandemic (parsimony and predictive capacity). According to this model, the units of analysis that experienced the least impact in the country were the Basque Country, La Rioja and Navarra. However, in each period, the relationships between the socioeconomic and epidemiological indicators varied, probably due to the actions of the public administrations. The COVID-19 pandemic is a complex phenomenon and certain aspects will be we will only be able to observe as years go by. However, more outcome variables such as excess mortality as well as the impact on the economy and citizens’ freedom should be considered.

## Figures and Tables

**Table 1:** Health system structure, epidemiological and socioeconomic indicators.

**Indicator**	Formula	Definition	Source of Information
CAM †ij	$\frac{\mathrm{Number}\;\mathrm{of}\;\mathrm{beds}\;\mathrm{before}\;\mathrm{COVID}-19}{\mathrm{Population}}$× 1.000	Bed rate	Ministry of Health, Consumer Affairs and Social Welfare
IE †ij	$\frac{\mathrm{Population}\;65+}{\mathrm{Population}\;0-15}$× 100	Ageing index ‡	National Statistics Institute (INE)
DEN †ij	$\frac{\mathrm{Population}}{\mathrm{Total}\;\mathrm{area}}$	Population density	National Statistics Institute (INE)
RAP †ij	$\frac{\mathrm{Healthcare}\;\mathrm{workers}\;\mathrm{in}\;\mathrm{primary}\;\mathrm{care}\;\mathrm{pre}\;\mathrm{COVID}-19}{\mathrm{Population}}$× 100.000	Ratio of primary care professionals	Ministry of Health, Consumer Affairs and Social Welfare
RAH †ij	$\frac{\mathrm{Healthcare}\;\mathrm{workers}\;\mathrm{in}\;\mathrm{hospitals}\;\mathrm{pre}\;\mathrm{COVID}-19}{\mathrm{Number}\;\mathrm{of}\;\mathrm{beds}\;\mathrm{pre}-\mathrm{COVID}-19}$	Ratio of hospital care professionals	Ministry of Health, Consumer Affairs and Social Welfare
IN ijt	$\frac{\mathrm{COVID}-19\;\mathrm{Cases}}{\mathrm{Population}}\;$× 100.000	Incidence	National Epidemiology Centre and the Carlos III Health Institute.
IG ijt	$\frac{\mathrm{COVID}-19\;\mathrm{Hospitalized}\;\mathrm{cases}}{\mathrm{COVID}-19\;\mathrm{Cases}}$× 100	Severity Index	National Epidemiology Centre and the Carlos III Health Institute.
HP ijt	$\frac{\mathrm{COVID}-19\;\mathrm{Hospitalized}\;\mathrm{cases}}{\mathrm{Population}}$× 100.000	Hospitalization rate among the population	National Epidemiology Centre and the Carlos III Health Institute.
SAT ijt	$\frac{\mathrm{COVID}-19\;\mathrm{Hospitalized}\;\mathrm{cases}}{\mathrm{Number}\;\mathrm{of}\;\mathrm{hospital}\;\mathrm{beds}\;\mathrm{pre}-\mathrm{COVID}-19}$× 100	Bed saturation	National Epidemiology Centre and the Carlos III Health Institute.
SATUCI ijt	$\frac{\mathrm{COVID}-19\;\mathrm{ICU}\;\mathrm{admitted}\;\mathrm{cases}}{\mathrm{Number}\;\mathrm{of}\;\mathrm{ICU}\;\mathrm{beds}\;\mathrm{pre}-\mathrm{COVID}-19}$× 100	ICU bed saturation	National Epidemiology Centre and the Carlos III Health Institute.
LET ijt	$\frac{\mathrm{COVID}-19\;\mathrm{Deaths}}{\mathrm{COVID}-19\;\mathrm{Cases}}$× 100	Lethality	National Epidemiology Centre and the Carlos III Health Institute.
MOR ijt	$\frac{\mathrm{COVID}-19\;\mathrm{Deaths}}{\mathrm{Population}}$× 100.000	Mortality	National Epidemiology Centre and the Carlos III Health Institute.
UCI ijt	$\frac{\mathrm{COVID}-19\;\mathrm{ICU}\;\mathrm{admitted}\;\mathrm{cases}}{\mathrm{COVID}-19\;\mathrm{Cases}}$× 100	Rate of ICU admissions among cases	National Epidemiology Centre and the Carlos III Health Institute.
EXMOR ijt	$\frac{\mathrm{Observed}\;\mathrm{deaths}-\mathrm{Expected}\;\mathrm{deaths}}{\mathrm{Population}}$× 100.000	Excess mortality	Daily Mortality Monitoring System (MoMo) and the Instituto de Salud Carlos III.
EXMOR_NOCOVID ijt	Observed deaths−COVID Deaths−Expected deaths		Daily Mortality Monitoring System (MoMo) and the Instituto de Salud Carlos III and National Epidemiology Centre and the Carlos III Health Institute.
EXES ij	$\frac{2020\;\mathrm{Waiting}\;\mathrm{list}-2019\;\mathrm{Waiting}\;\mathrm{list}}{\mathrm{Population}}$× 100.000	Excess of patients on the waiting list	Ministry of Health, Consumer Affairs and Social Welfare
CASOS ijt	-	COVID-19 cases	National Epidemiology Centre and the Carlos III Health Institute.
PIB ij	-	Debt % GDP	Macro Expansion Data
CAP ij	-	Debt per capita	Macro Expansion Data
PARO ijt	$\frac{\mathrm{Number}\;\mathrm{of}\;\mathrm{unemployed}\;\mathrm{persons}}{\mathrm{Number}\;\mathrm{of}\;\mathrm{working}\;\mathrm{persons}}$	Unemployment rate	National Statistics Institute (INE)
ERTE ijt	Average number of people in ERTE in each quarter	Persons in ERTE	General Treasury of the Social Security (TGSS)
EMSS ijt	$\frac{\mathrm{Companies}\;\mathrm{in}\;\mathrm{SS}\;p*\;\mathrm{Companies}\;\mathrm{in}\;\mathrm{SS}\;p-1}{\mathrm{Companies}\;\mathrm{in}\;\mathrm{SS}\;p-1}$× 100	Percentage variation of companies registered in the Social Security system	Ministry of Labour and Social Economy
NEM ijt	$\frac{\mathrm{New}\;\mathrm{companies}\;p\;-\mathrm{New}\;\mathrm{companies}\;p-1}{\mathrm{New}\;\mathrm{companies}\;p-1}$× 100	Percentage variation of new companies created	National Statistics Institute (INE)

† Pre COVID-19 health system structure indicators (data as of 1 January 2020); i All the variables were calculated for each of the i-autonomous communities; j All variables were calculated for the year 2020; t All variables were calculated for each of the quarters of 2020 and for quarter 1 of 2020; ‡ The Ageing Index (AI) is defined as the quotient between the number of persons over 64 years of age and the population under 16 years of age on January 1st of year t. (INE)**;** * Period *p* corresponds to quarter x of the year 2020 and period *p*−1 corresponds to quarter x of the year 2019.

**Table 2:** Correlations between socioeconomic and epidemiological indicators. 2020 data.

	IN	IG	HP	SAT	SATUCI	LET	MOR	UCI	CASOS	EXMOR	EXMOR_ NOCOVID
IE	−0.15	0.55 *	0.13	−0.09	−0.21	0.67 *	0.30	0.19	−0.19	0.00	−0.24
PIB	−0.04	−0.28	−0.18	−0.14	−0.22	−0.09	−0.07	−0.32	0.16	0.04	0.15
CAP	0.19	−0.09	0.10	0.06	−0.04	0.08	0.19	−0.30	0.41	0.01	0.27
PARO	−0.49 *	−0.34	−0.51 *	−0.33	−0.38	−0.43	−0.54 *	0.20	−0.07	−0.18	0.09
ERTE	−0.16	−0.02	−0.06	0.12	−0.19	−0.14	−0.16	−0.06	0.80 *	−0.09	0.68 *
EMSS	0.59 *	0.22	0.53 *	0.35	0.31	0.39	0.56 *	−0.44	0.18	0.27	0.09
NEM	−0.41	−0.17	−0.40	−0.34	−0.39	−0.19	−0.46	−0.05	−0.05	−0.47 *	−0.08

* The highlighted boxes indicate correlations with a significance < 0.05; EI: aging index; GDP: debt %; CAP: debt per capita; PARO: unemployment rate; ERTE: number of persons in ERTE; EMSS: percentage change in companies registered with Social Security; NEM: percentage change in new companies created; IN: incidence; IG: severity index; HP: hospitalization-to-population ratio; SAT: bed saturation; SATUCI: saturation of ICU beds; LET: lethality; MOR: mortality; UCI: rate of ICU admissions among cases; CASOS: COVID-19 cases; EXMOR: excess mortality; EXMOR_NOCOVID: excess mortality theoretically due to causes other than COVID-19.

**Table 3:** Canonical loads for each of the models were performed.

**Epidemiologic Indicators**						
	Model Q1 2020	Model Q2 2020	Model Q3 2020	Model Q4 2020	Model anual 2020	Model Q1 2021
**IN**	−0.20	−1.98	−0.72	−1.71	−1.27	0.52
**SAT**	−1.21	0.22	0.21	0.13	−0.01	0.44
**LET**	−0.21	−0.10	−1.07	−1.77	−0.93	0.47
**UCI**	0.09	−0.20	−0.11	−0.28	−0.03	−0.39
**EXMOR**	1.58	1.04	1.16	2.17	1.17	−1.43
**Socioeconomic Indicators**						
	Model Q1 2020	Model Q2 2020	Model Q3 2020	Model Q4 2020	Model anual 2020	Model Q1 2021
**PARO**	0.09	1.11	0.64	0.97	0.72	−0.72
**EMSS**	−0.60	0.11	0.66	−0.05	−0.32	0.37
**NEM**	−0.60	0.23	0.73	−0.16	0.25	0.26

IN: incidence; SAT: bed saturation; LET: lethality; ICU: rate of COVID-19 admissions among COVID-19 cases; EXMOR: excess mortality; PARO: unemployment rate; EMSS: percentage change in companies registered with Social Security; NEM: percentage change in new companies created.

## Data Availability

The datasets generated and/or analysed during the current study are available in the OSF repository, https://doi.org/10.17605/OSF.IO/5PV28.

## Supplementary Materials

The following supporting information can be downloaded at: https://www.mdpi.com/article/10.3390/ijerph192113981/s1, Table S1. Different measures adopted in each autonomous community during December 2020; Table S2. Mortality excess during the COVID-19 pandemic in the Autonomous Communities. (ratios); Table S3. Evolution of the incidence and mortality of COVID-19 by Autonomous Community; Table S4. Evolution of the economic situation by Autonomous Community; Table S5. Correlations between socioeconomic and epidemiological indicators. Data quarter 1 2020; Table S6. Correlations between socioeconomic and epidemiological indicators. Data quarter 1 2021; Figure S1. Correlations of all socioeconomic and epidemiological variables of annual 2020 dataset; Figure S2. Correlations of all socioeconomic and epidemiological variables of Q1 2020 dataset; Figure S3. Correlations of all socioeconomic and epidemiological variables of Q2 2020 dataset; Figure S4. Correlations of all socioeconomic and epidemiological variables of Q3 2020 dataset; Figure S5. Correlations of all socioeconomic and epidemiological variables of Q4 2020 dataset; Figure S6. Correlations of all socioeconomic and epidemiological variables of T1 2020 dataset; Graphical representation of the canonical correlations in each of the analysis periods.

## Abbreviations

NPIs: non-pharmaceutical interventions; GDP: Gross Domestic Product; RENAVE: Red Nacional de Vigilancia Epidemiológica; INE: Instituto Nacional de Estadística; EI: ageing index; GDP: debt %; CAP: debt per capita; PARO: unemployment rate; ERTE: number of persons in ERTE; EMSS: percentage change in companies registered with Social Security; NEM: percentage change in new companies created; IN: incidence; IG: severity index; HP: hospitalization-to-population ratio; SAT: bed saturation; SATUCI: saturation of ICU beds; LET: lethality; MOR: mortality; UCI: rate of ICU admissions among cases; CASOS: COVID-19 cases; EXMOR: excess mortality; EXMOR_NOCOVID: excess mortality theoretically due to causes other than COVID-19. AN: Andalusia; AR: Aragon; AS: Asturias; IB: Balearic Islands; CN: Canary Islands; CB: Cantabria; CM: Castilla La-Mancha; CL: Castilla y León; CT: Catalonia; CEyML: Ceuta and Melilla; VC: Community of Valencia; EX: Extremadura; GA: Galicia; RI: La Rioja; MD: Community of Madrid; MC: Murcia; NC: Navarra; PV: Basque Country.
