# Supplementary material for: Measuring the Impact of Future Outbreaks? A Secondary Analysis of Routinely Available Data in Spain
Source: Int J Environ Res Public Health. 2022 Oct 27;19(21):13981. doi: 10.3390/ijerph192113981 (PMC9655530; doi:10.3390/ijerph192113981)
Supplement: Supplementary file 1 [file ijerph-19-13981-s001.zip › ijerph-1985138-supplementary.pdf]

Table S1. Different measures adopted in each autonomous community during December 2020.

Autonomous Community	Value	C (perimeter limitation of the region)	D (perimeter limitation between municipalities)	E (curfew)	F (limitation of social groups)	Official Source	Website	Date
Canary Islands	A + C + D	Yes, the entry restriction will not apply to those passengers who undergo the health control consisting of: subscription of a responsible declaration, symptomatology control or diagnostic test for Active Infection with negative result in the 72 hours prior to arrival and/or isolation.	No limitation.	Between 00:00h and 06:00h: La Palma and La Gomera. Between 23:00h and 06:00h: Tenerife, Gran Canaria, Lanzarote, Fuerteventura and El Hierro.	Maximum 10 people: La Palma, La Gomera. Maximum 6 people, except cohabitants: Tenerife, Gran Canaria, Lanzarote, Fuerteventura and El Hierro.	BOC	http://www.gobiernodecanarias.org/boc/	-
Melilla	A + C + D	Yes.	No limitation.	Between 22:00 and 6:00 hours.	Maximum 4 people in public spaces, both closed and open. Only cohabitants in private spaces.	BOME	http://www.melilla.es/melillaportal/conten	27/10/2020

							edor.jsp?seccion=bome.jsp	
Ceuta	A + C + D	Yes.	No limitation.	Between 00:00 and 5:00 hours.	Maximum 4 people in public spaces, both indoors and outdoors and in the private sphere.	BOCCE	http://www.ceuta.es/ceuta/documentos/	29/10/2020
La Rioja	A + B + C + D	Yes.	Entry and exit is limited to the municipalities of: Calahorra, Alfaro, Arnedo and Nájera.	Between 23:00h and 05:00h. Between 22:00h and 06:00h for the municipalities of Calahorra, Alfaro, Arnedo and Nájera.	Maximum 6 people. Only people living together in Cervera del Río Alhama. Maximum 4 people, except for people living together in Calahorra, Nájera, Alfaro, Arnedo and Rincón de Soto.	BOR	http://www.larioja.org/bor/es	29/10/2020
Basque Country	A + B + C + D	Yes.	See list of municipalities with limitations at: https://www.euskadi.eus/coronavirus/	Between 22:00 and 6:00 hours.	Maximum 4 people in public (indoor and outdoor) and private spaces.	BOPV	https://www.euskadi.eus/y22-bopv/es/bopv2/datos/Ultimo.shtml	26/10/2020

Autonomous Community of Navarre	A + B + C + D	Yes.	Limitation of the entry and exit of the municipalities of: Etxarria Arantz and Lakuntza.	Between 23:00 and 6:00 hours.	Maximum 4 people in enclosed public spaces and 6 in open public spaces. Only cohabitants in private spaces.	BON	http://www.navarra.es/home_es/Actualidad/BON/	3/11/2020
Region of Murcia	A + C + D	Yes.	No limitation.	Between 00:00 and 6:00 hours.	Maximum 6 people in open public spaces. Maximum 4 people in closed and private public spaces.	BORM	https://www.borm.es/#/home/	30/10/2020

Community of Madrid	B + C + D	No limitation	Limitation of entry and exit in: Arganzual (La Chopera), Barajas (Barajas), Ciudad lineal (Gandhi, Daroca), Latina (General Fanjul), Hortaleza (silvano, Vicente Muzas), Salamanca (Castelló), Villa de Vallecas, Getafe (Las Margaritas, Las Ciudades), Móstoles (La princesa, Alcalde Bartolome González), Majadahonda (majadahonda), Las Rozas (Las Rozas), San Sebastian de Los Reyes (Reyes Catolicos), Manzanares El Real and San Agustin de Guadalix. < br> From May 9 in Madrid: Ciudad Lineal (Daroca), Hortaleza	Between 23:00 and 6:00 hours.	In open public spaces limited to a maximum of 6 people and in closed public spaces up to a maximum of 4 people. In private spaces it is limited to the nucleus of coexistence.	BOCM	http://www.bocm.es/	29/10/2020

			(Vicente Muzas), Getafe (Las Ciudades), Las Rozas de Madrid (Las Rozas), San Sebastian de Los Reyes (Reyes Catolicos).					

Galicia	A + B + C + D	Yes.	Specific limitations of entry and exit of people and permanence of groups of people in public or private spaces in Cambados, Cualedro, Laza and Vilanova de Arousa.	Between 23:00 and 6:00 hours.	Maximum 4 people in enclosed spaces for public use and six in open spaces or outdoors for public use. In spaces for private use, the stay of groups of people will be limited exclusively to cohabitants. In municipalities with perimeter restrictions, only groups of people living together are allowed.	DOG	https://www.xunta.gal/diario-oficial-galicia/portalPublicoHome.do?lang=es	21/10/2020
Extremadura	B + C + D	No limitation	Limitation of the entrance and exit of the municipalities of: Guadalupe, Segura de León, Oliva de Mérida, Zarza de Granadilla, Guijo de Granadilla, Ahigal, Piornal, Palomas and Bodonal de la Sierra.	Between 00:00 and 6:00 hours.	Maximum 4 people in enclosed public spaces and 6 in open public spaces. These numerical limitations do not apply in the case of groups of cohabitants.	DOE	http://doe.gobex.es/	28/10/2020

Valencian Community	A + C + D	Yes.	No limitation.	Between 22:00 and 6:00 hours.	In open and closed public spaces, maximum of 6 people. In the private area maximum two cohabitation groups.	DOGV	http://www.dogv.gva.es/es/	31/10/2020
Catalonia	A + C + D	Yes.	No limitation.	Between 22.00 and 06.00 hours.	Family and social gatherings and/or meetings, both public and private, are permitted as long as they do not exceed the maximum number of six people, except in the case of cohabitants.	DOGC	https://dogc.gencat.cat/es/index.html?newLang=es_ES&language=es_ES	30/10/2020
Castilla-La Mancha	A + C + D	Yes.	No limitation.	Between 00:00 and 06.00 hours.	Maximum 6 people.	DOCM	http://docm.castillalamancha.es/portaldocm/sumario.do	29/10/2020
Castilla y León	A + C + D	Yes.	No limitation.	Between 22:00 and 6:00 hours.	**From March 26th to April 9th:** Maximum 4 people in public spaces and only cohabitants in private spaces.	BOCYL	http://bocyl.jcyl.es/	29/10/2020

Cantabria	A + C + D	Yes.	No limitation.	Between 22:00 and 6:00 hours.	Maximum 4 people.	BOC	https://boc.cantabria.es/boces/	29/10/2020
Balearic Islands	A + C + D	Yes.< b>Until 9 May: Persons arriving in the Balearic Islands must undergo a check on the reason for the journey and, where appropriate, a health check prior to entry.	No limitation.	Between 23:00 and 6:00 hours.	Maximum 6 people. In the interior only meetings of a maximum of two people are allowed. On the islands of Mallorca and Ibiza: maximum four people outside. On the islands of Menorca and Formentera: maximum four people inside, as long as they belong to a maximum of two cohabitation groups, except if they are cohabitants, and six people outside.	BOIB	http://www.caib.es/eboibfront/?lang=es	29/10/2020
Principality of Asturias	A + B + C + D	Yes.	Restriction of the entrance and exit of the council of Llanes.	Between 22:00 and 6:00 hours.	Maximum 6 people. In the confined municipalities, it is limited to a maximum of 4 persons. Meetings in private homes are	BOPA	http://sede.asturias.es/bopa	29/10/2020

					limited to the persons living together.			
Aragon	A + B + C + D	Yes.	Limitation of the entry and exit of the municipalities of: Tarazona, Jaca and Calatayud. Regions of: Cinco Villas, Campo de Cariñena, Ribera Alta del Ebro and Valdejalón.	Between 23:00 and 6:00 hours.	Maximum 4 people in enclosed public spaces and 6 in open public spaces, except in the case of cohabitants. In private spaces, meetings shall be limited to cohabitants. In confined municipalities, groups of people in public and private spaces shall be limited to a maximum of four people, unless they are cohabitants.	BOA	http://www.boa.aragon.es/	26/10/2020
Andalusia	A + C + D	Yes.	Mobility between all provinces is allowed. However, entry and exit is restricted for municipalities with more than 500 Cumulative Incidence	Between 23:00 and 6:00 hours.	Maximum 6 people.	BOJA	http://www.juntadeandalucia.es/eboja	30/10/2020

			cases per 100,000 inhabitants in 14 days.					

Table S2. Mortality excess during the COVID-19 pandemic in the Autonomous Communities. (ratios)

CCAA	Q1 2020	Q2 2020	Q3 2020	Q4 2020	Annual 2020	Q1 2021
Andalucía	7	11	22	50	89	35
Aragón	18	40	50	77	186	20
Asturias	17	42	16	100	175	21
Islas Baleares	-5	-3	15	17	24	7
Canarias	4	-4	10	0	11	-12
Cantabria	2	22	7	16	47	-27
Castilla y León	49	94	23	76	241	10
Castilla La-Mancha	109	135	25	44	313	23
Cataluña	41	94	9	37	181	11
Ceuta y Melilla	74	29	40	81	224	62
Comunidad Valenciana	4	18	20	31	73	65
Extremadura	29	53	36	54	172	43
Galicia	3	2	13	16	35	3
La Rioja	41	66	31	60	198	36
Madrid	103	100	21	18	243	22
Murcia	-2	7	17	28	50	31
Navarra	45	58	13	45	160	-12
País Vasco	20	43	19	35	117	-1

Cases per 100,000 inhabitants

Table S3. Evolution of the incidence and mortality of COVID-19 by Autonomous Community.

CCAA	Q1 2020		Q2 2020		Q3 2020		Q4 2020		ANNUAL 2020		Q1 2021	
	IN	MOR	IN	MOR	IN	MOR	IN	MOR	IN	MOR	IN	MOR
Andalucía	92	5	68	12	640	6	2326	39	3127	62	2817	48
Aragón	196	16	277	53	2239	37	3269	90	5982	197	2277	54
Asturias	135	7	104	26	302	2	2137	100	2678	135	1975	52
Islas Baleares	106	5	91	14	1039	11	1905	16	3141	46	1792	23
Canarias	68	3	43	4	499	4	610	8	1219	20	890	11
Cantabria	212	10	194	26	768	3	1.914	30	3088	70	1397	23
Castilla y León	333	33	539	86	1208	12	3299	86	5380	217	3240	58
Castilla La-Mancha	395	54	532	90	1366	12	2870	55	5163	213	3460	68
Cataluña	290	26	427	58	1066	8	2813	43	4595	135	2283	43
Ceuta y Melilla	67	1	103	3	819	6	3479	52	4467	62	3158	41
Comunidad Valenciana	130	10	110	19	609	4	2519	31	3368	64	4235	80
Extremadura	158	16	148	32	722	8	2510	45	3538	101	3084	62
Galicia	184	5	163	18	456	5	1487	24	2289	52	2031	34
La Rioja	669	34	590	81	1417	18	3095	51	5771	184	2973	49
Madrid	610	59	481	70	2565	17	2344	31	6000	177	3297	39
Murcia	71	3	48	6	1240	5	2696	36	4055	51	3134	53
Navarra	382	23	467	57	1959	9	3637	59	6446	148	1796	21
País Vasco	315	20	351	50	1576	16	3058	51	5300	137	2081	45

IN: incidence (cases per 100,000 inhabitants)

MOR: mortality (deaths per 100,000 inhabitants)

Table S4. Evolution of the economic situation by Autonomous Community

CCAA	Q1 2020*		Q2 2020		Q3 2020		Q4 2020		ANNUAL 2020		Q1 2021	
	EMSS	PARO	EMSS	PARO	EMSS	PARO	EMSS	PARO	EMSS	PARO	EMSS	PARO
Andalucía	-4.4	21.2	-8.1	21.3	-3.4	23.8	-2.2	22.7	-4.5	22.7	1.6	22.5
Aragón	-1.9	10.6	-4.4	11.8	-2.9	11.9	-3.1	12.5	-3.1	12.5	-1.8	12.1
Asturias	-2.9	14.4	-6.4	14.5	-2.9	14.2	-3.2	13.5	-3.9	13.5	-1.7	14.1
Islas Baleares	-1.6	18.2	-10.1	15.9	-7.1	13.3	-3.9	17.3	-5.9	17.3	-4.6	18.9
Canarias	-3.5	18.8	-8.8	21.6	-5.5	25	-5.8	25.2	-5.9	25.2	-3.8	25.4
Cantabria	-1.8	11.1	-5.9	13.8	-2.9	12	-2.7	11.8	-3.3	11.8	-1.8	11.9
Castilla y León	-2.4	11.8	-5.8	12.4	-3.4	12.5	-3.7	11.6	-3.8	11.6	-2.6	12.6
Castilla La-Mancha	-3.6	18.1	-5.5	16.8	-2.3	18.3	-1.5	17.4	-3.2	17.4	0.3	17.4
Cataluña	-1.7	10.7	-6	12.8	-3.3	13.2	-3.2	13.9	-3.6	13.9	-1.5	12.9
Ceuta y Melilla	-4.4	23.5	-6.2	21.8	-4.7	25.6	-7.4	25.3	-5.7	25.5	-5.2	25
Comunidad Valenciana	-2.1	14.4	-6.6	16.7	-2.9	17.3	-2.7	16.4	-3.6	16.4	-2.5	16.5
Extremadura	-2.7	23.6	-5.9	21.4	-2.9	20.9	-2.6	21.3	-3.5	21.3	0	22.2
Galicia	-1.9	12.7	-5.5	12	-3.4	11.8	-3.1	11.7	-3.5	11.7	-2.2	13
La Rioja	-2.3	11.2	-5	10.1	-2.5	11.5	-2.6	10.4	-3.1	10.4	-2.4	11.9
Madrid	-1.8	10.6	-5.4	12.6	-2.7	13.3	-3	13.5	-3.2	13.5	-1	12.2
Murcia	-2.2	16.5	-5.4	15.6	-2.1	17.2	-2.7	15.4	-3.1	15.4	-1.1	16.4
Navarra	-1.4	8.6	-3.8	10.1	-2.1	9.9	-2.5	11.7	-2.4	11.7	-1.1	11.5
País Vasco	-1.4	8.7	-3.6	9.1	-1.9	10.3	-2.3	10	-2.3	10	-1.4	11

EMSS: percentage change in the number of companies registered with Social Security
PARO: unemployment rate (%)

Table S5. Correlations between socioeconomic and epidemiological indicators. Data quarter 1 2020

	IN	IG	HP	SAT	SATUCI	LET	MOR	UCI	CASOS	EXMOR	EXMOR_NOCOVID
PARO	-0.57	0.03	-0.45	-0.35	-0.51	-0.08	-0.37	0.37	-0.32	-0.01	-0.2
ERTE	0.11	0.43	0.30	0.34	0.33	0.27	0.24	0.16	0.77	0.11	0.56
EMSS	0.35	-0.04	0.27	0.20	0.35	0.07	0.18	-0.17	0.21	-0.21	0.08
NEM	-0.04	0.30	0.10	0.14	0.14	0.12	0.03	0.37	0.48	-0.30	0.27

*The highlighted boxes indicate correlations with a significance < 0.05.

PARO: unemployment rate; EMSS: percentage change in companies registered with Social Security; NEM: percentage change in new companies created; IN: incidence; IG: severity index; HP: hospitalization rate among population; SAT: bed saturation; SATUCI: ICU bed saturation; LET: case fatality; MOR: mortality; UCI: rate of ICU admissions among cases; EXMOR: excess mortality; EXMOR_NOCOVID: excess mortality theoretically due to causes other than COVID-19

Table S6. Correlations between socioeconomic and epidemiological indicators. Data quarter 1 2021

	IN	IG	HP	SAT	SATUCI	LET	MOR	UCI	CASOS	EXMOR	EXMOR_NOCOVID
PARO	0.05	0.07	-0.22	0.02	0.17	-0.27	-0.06	0.17	0.00	0.16	0.26
ERTE	0.06	-0.38	-0.21	0.06	-0.42	-0.15	-0.02	-0.32	0.82	0.03	-0.81
EMSS	0.24	0.27	0.12	0.13	-0.42	0.34	0.37	-0.47	0.45	0.40	-0.33
NEM	-0.28	-0.19	-0.09	-0.08	-0.48	0.08	-0.15	0.12	0.05	0.06	0.00

*The highlighted boxes indicate correlations with a significance < 0.05.

PARO: unemployment rate; EMSS: percentage change in companies registered with Social Security; NEM: percentage change in new companies created; IN: incidence; IG: severity index; HP: hospitalization rate among population; SAT: bed saturation; SATUCI: ICU bed saturation; LET: case fatality; MOR: mortality; UCI: rate of ICU admissions among cases; EXMOR: excess mortality; EXMOR_NOCOVID: excess mortality theoretically due to causes other than COVID-19

Figure S1. Correlations of all socioeconomic and epidemiological variables of annual 2020 dataset.

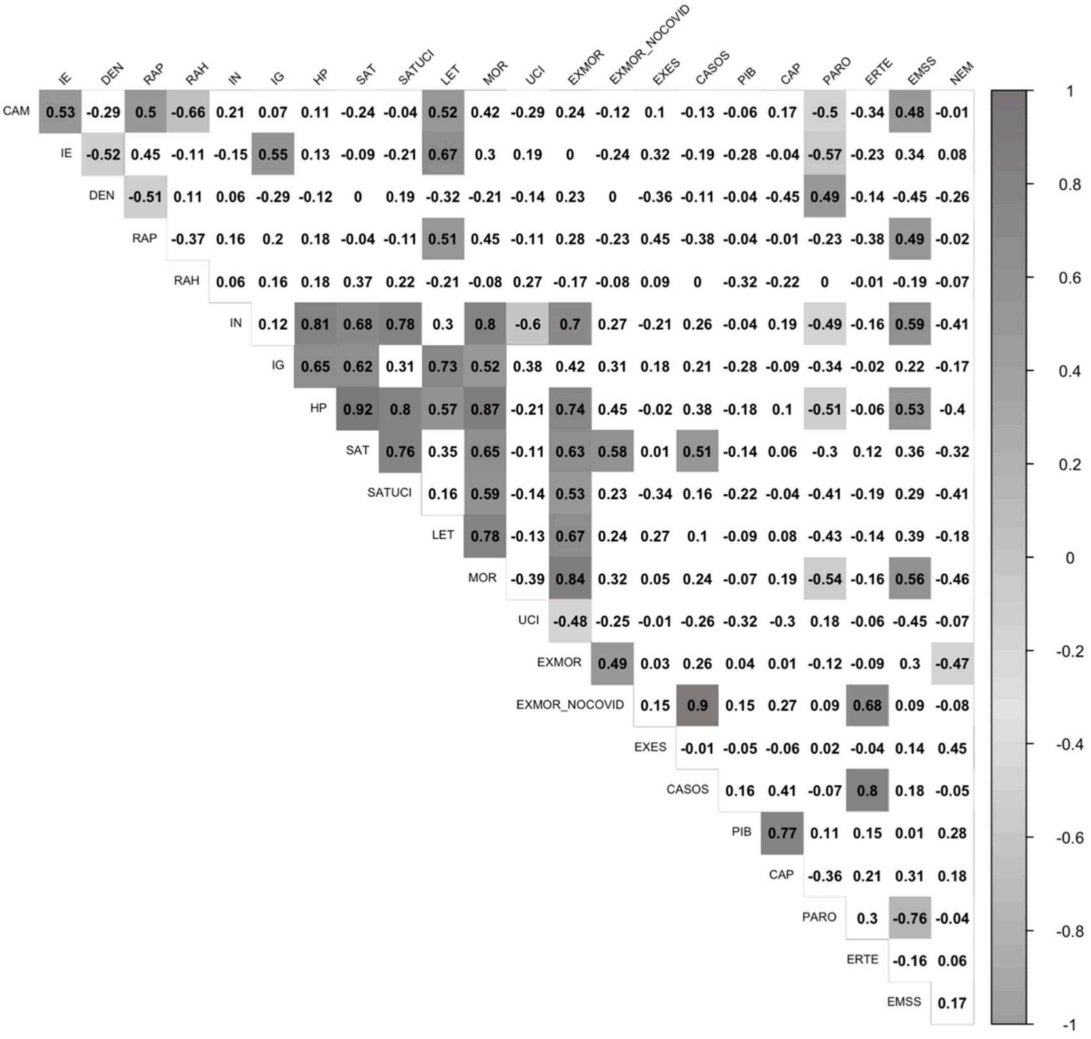

*The highlighted boxes reflect those correlations that have a significance < 0.05.

CAM: bed rate; IE: ageing index; DEN: population density; RAP: ratio of primary care professionals; RAH: Ratio of hospital care professionals; IN: incidence; IG: severity index; HP: hospitalization rate among population; SAT: bed saturation; SATUCI: ICU bed saturation; LET: case fatality; MOR: mortality; UCI: rate of ICU admissions among cases; EXMOR: excess mortality; EXMOR_NOCOVID: exceso de mortalidad de pacientes no COVID-19; EXES: excess of patients on waiting list; CASOS: COVID-19 cases; PIB: debt % of GDP; CAP: debt per capita; PARO: unemployment rate; ERTE: número de personas en ERTE; EMSS: percentage change in enterprises registered with Social Security; NEM: percentage change in new enterprises created.

Figure S2. Correlations of all socioeconomic and epidemiological variables of T1 2020 dataset.

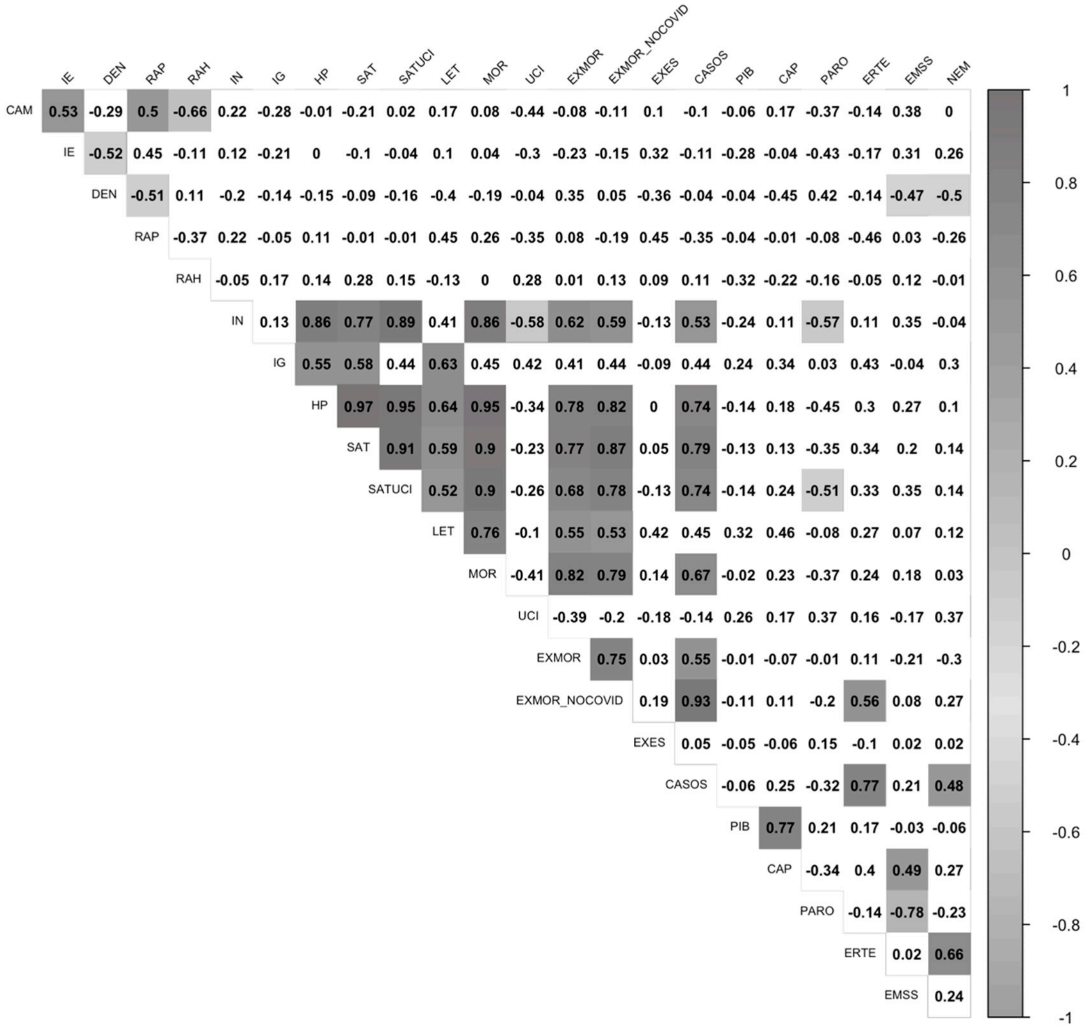

*The highlighted boxes reflect those correlations that have a significance < 0.05.

CAM: bed rate; IE: ageing index; DEN: population density; RAP: ratio of primary care professionals; RAH: Ratio of hospital care professionals; IN: incidence; IG: severity index; HP: hospitalization rate among population; SAT: bed saturation; SATUCI: ICU bed saturation; LET: case fatality; MOR: mortality; UCI: rate of ICU admissions among cases; EXMOR: excess mortality; EXMOR_NOCOVID: exceso de mortalidad de pacientes no COVID-19; EXES: excess of patients on waiting list; CASOS: COVID-19 cases; PIB: debt % of GDP; CAP: debt per capita; PARO: unemployment rate; ERTE: número de personas en ERTE; EMSS: percentage change in enterprises registered with Social Security; NEM: percentage change in new enterprises created

Figure S3. Correlations of all socioeconomic and epidemiological variables of T2 2020 dataset.

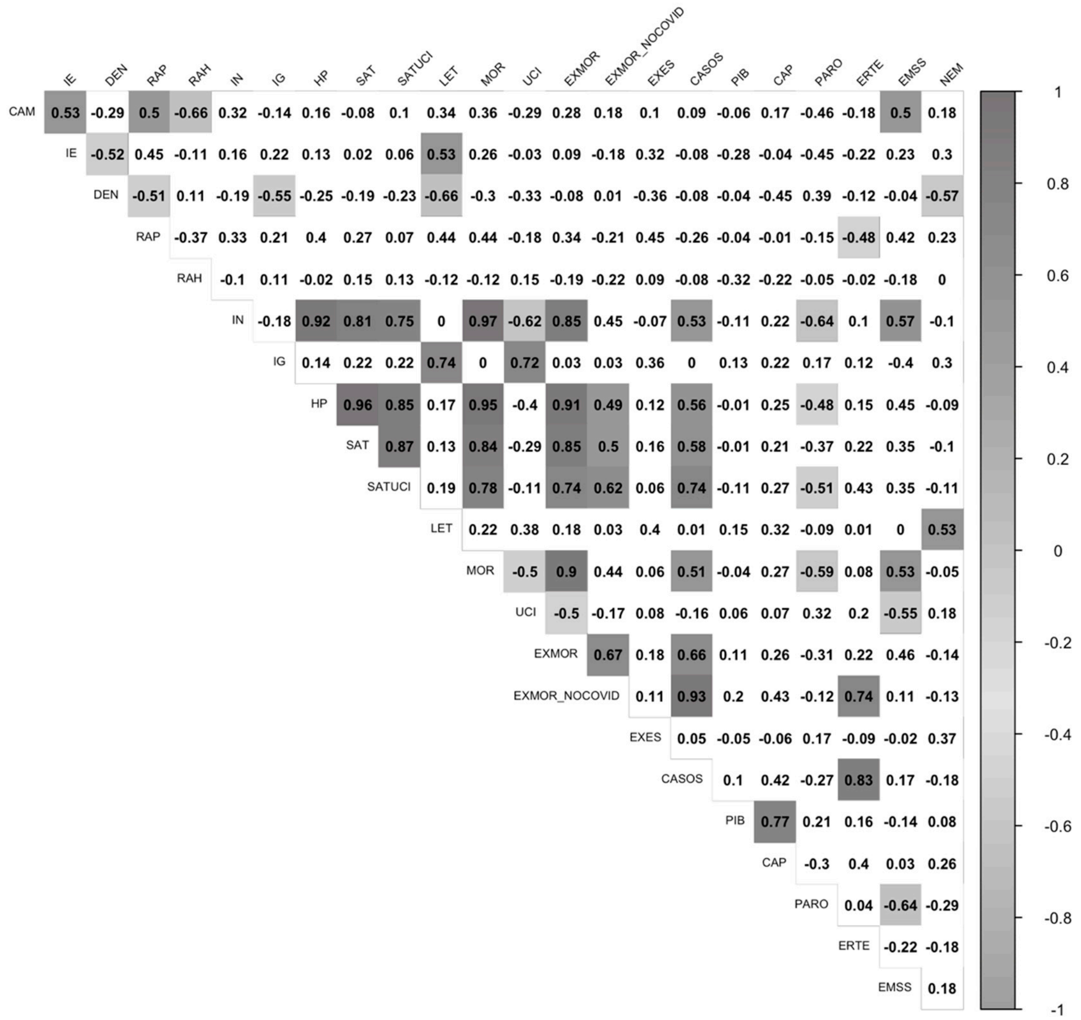

*The highlighted boxes reflect those correlations that have a significance < 0.05.

CAM: bed rate; IE: ageing index; DEN: population density; RAP: ratio of primary care professionals; RAH: Ratio of hospital care professionals; IN: incidence; IG: severity index; HP: hospitalization rate among population; SAT: bed saturation; SATUCI: ICU bed saturation; LET: case fatality; MOR: mortality; UCI: rate of ICU admissions among cases; EXMOR: excess mortality; EXMOR_NOCOVID: exceso de mortalidad de pacientes no COVID-19; EXES: excess of patients on waiting list; CASOS: COVID-19 cases; PIB: debt % of GDP; CAP: debt per capita; PARO: unemployment rate; ERTE: número de personas en ERTE; EMSS: percentage change in enterprises registered with Social Security; NEM: percentage change in new enterprises created

Figure S4. Correlations of all socioeconomic and epidemiological variables of T3 2020 dataset.

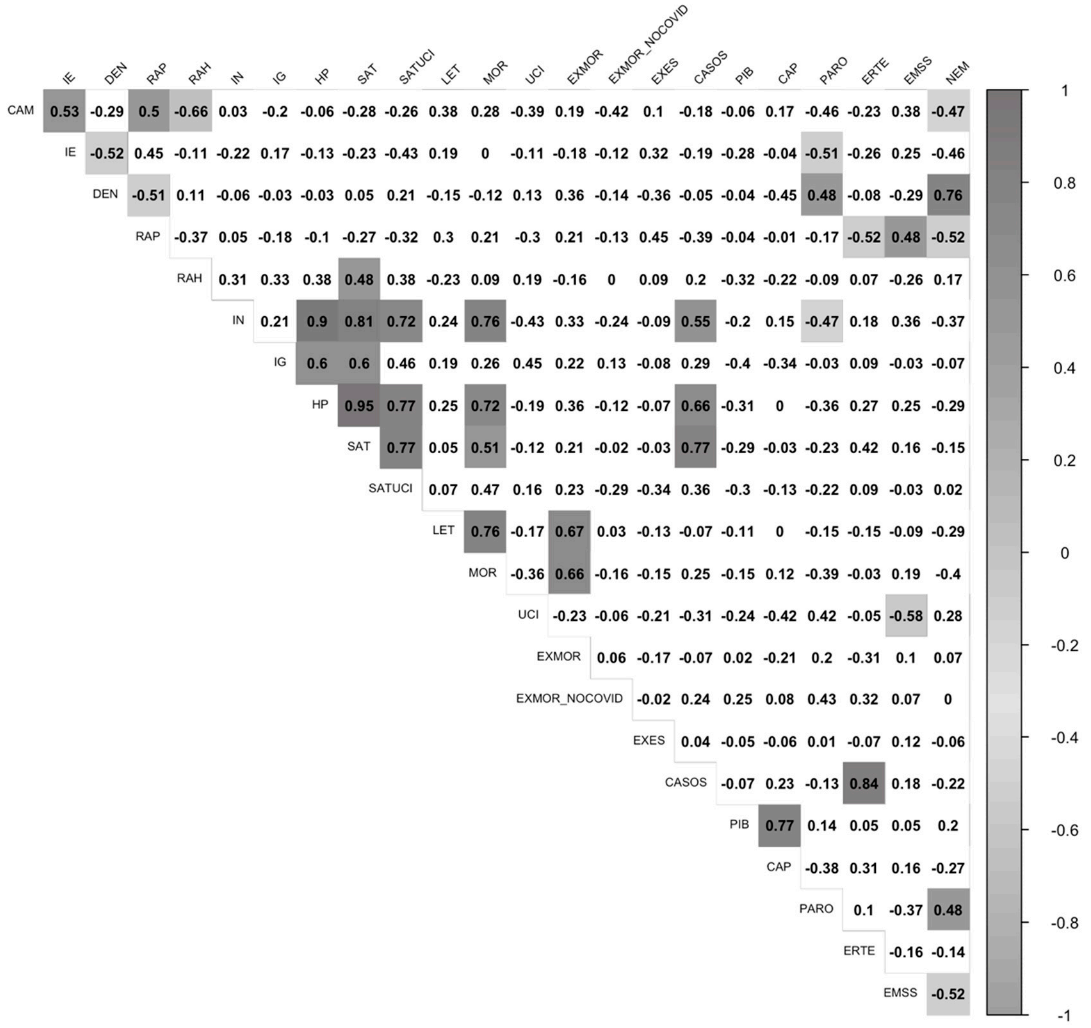

*The highlighted boxes reflect those correlations that have a significance < 0.05.

CAM: bed rate; IE: ageing index; DEN: population density; RAP: ratio of primary care professionals; RAH: Ratio of hospital care professionals; IN: incidence; IG: severity index; HP: hospitalization rate among population; SAT: bed saturation; SATUCI: ICU bed saturation; LET: case fatality; MOR: mortality; UCI: rate of ICU admissions among cases; EXMOR: excess mortality; EXMOR_NOCOVID: exceso de mortalidad de pacientes no COVID-19; EXES: excess of patients on waiting list; CASOS: COVID-19 cases; PIB: debt % of GDP; CAP: debt per capita; PARO: unemployment rate; ERTE: número de personas en ERTE; EMSS: percentage change in enterprises registered with Social Security; NEM: percentage change in new enterprises created

Figure S5. Correlations of all socioeconomic and epidemiological variables of T4 2020 dataset.

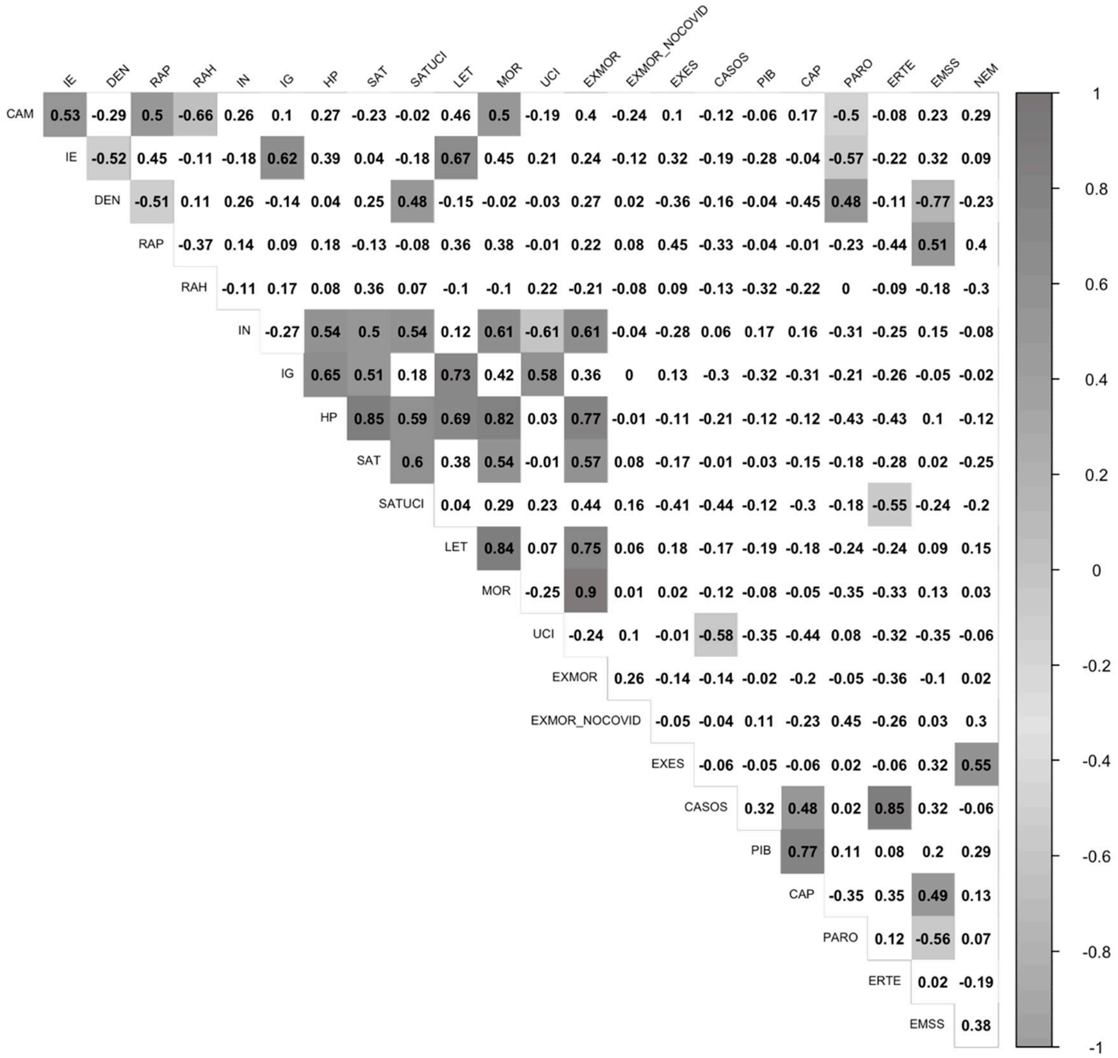

*The highlighted boxes reflect those correlations that have a significance < 0.05.

CAM: bed rate; IE: ageing index; DEN: population density; RAP: ratio of primary care professionals; RAH: Ratio of hospital care professionals; IN: incidence; IG: severity index; HP: hospitalization rate among population; SAT: bed saturation; SATUCI: ICU bed saturation; LET: case fatality; MOR: mortality; UCI: rate of ICU admissions among cases; EXMOR: excess mortality; EXMOR_NOCOVID: exceso de mortalidad de pacientes no COVID-19; EXES: excess of patients on waiting list; CASOS: COVID-19 cases; PIB: debt % of GDP; CAP: debt per capita; PARO: unemployment rate; ERTE: número de personas en ERTE; EMSS: percentage change in enterprises registered with Social Security; NEM: percentage change in new enterprises created

Figure S6. Correlations of all socioeconomic and epidemiological variables of T1 2020 dataset.

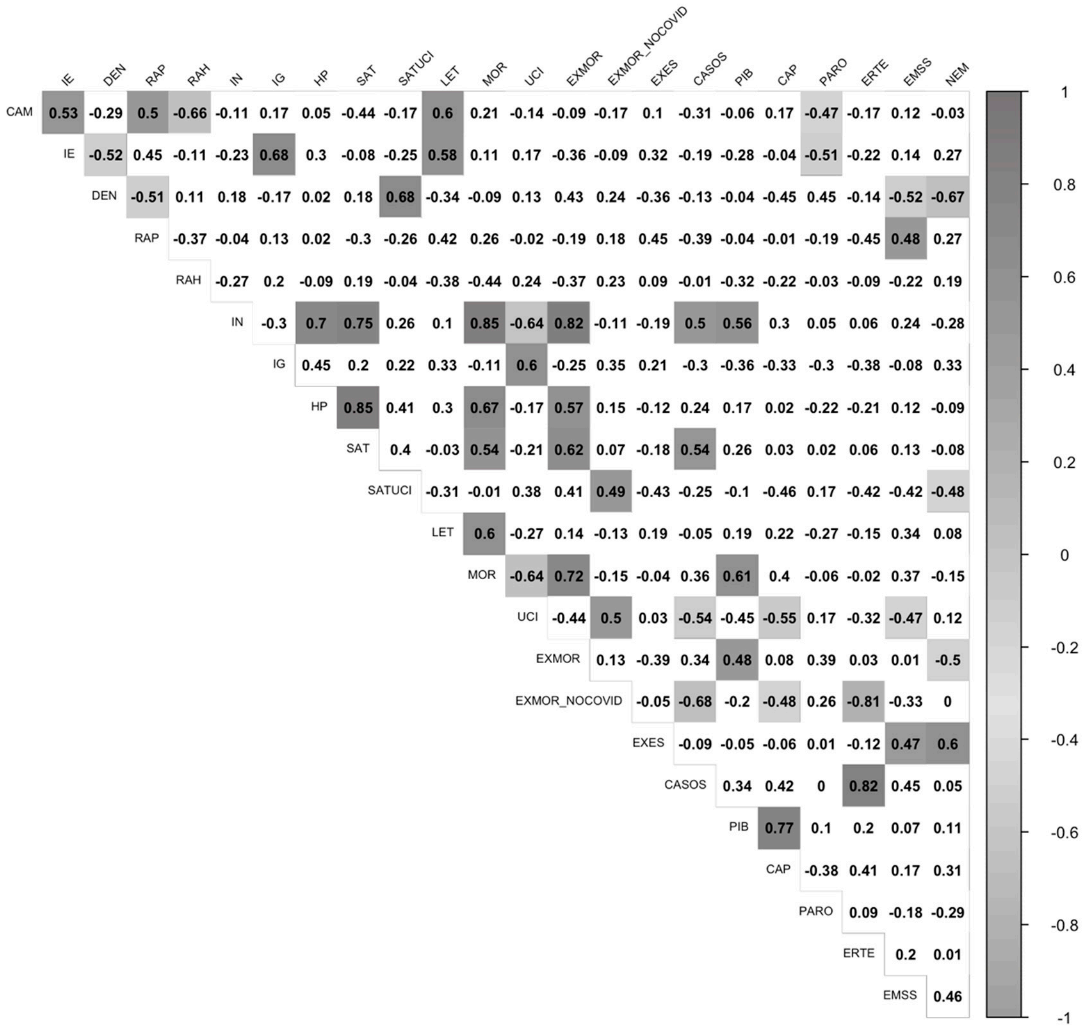

*The highlighted boxes reflect those correlations that have a significance < 0.05.

CAM: bed rate; IE: ageing index; DEN: population density; RAP: ratio of primary care professionals; RAH: Ratio of hospital care professionals; IN: incidence; IG: severity index; HP: hospitalization rate among population; SAT: bed saturation; SATUCI: ICU bed saturation; LET: case fatality; MOR: mortality; UCI: rate of ICU admissions among cases; EXMOR: excess mortality; EXMOR_NOCOVID: exceso de mortalidad de pacientes no COVID-19; EXES: excess of patients on waiting list; CASOS: COVID-19 cases; PIB: debt % of GDP; CAP: debt per capita; PARO: unemployment rate; ERTE: número de personas en ERTE; EMSS: percentage change in enterprises registered with Social Security; NEM: percentage change in new enterprises created

Graphical representation of the canonical correlations in each of the analysis periods.

Period Q1-2020

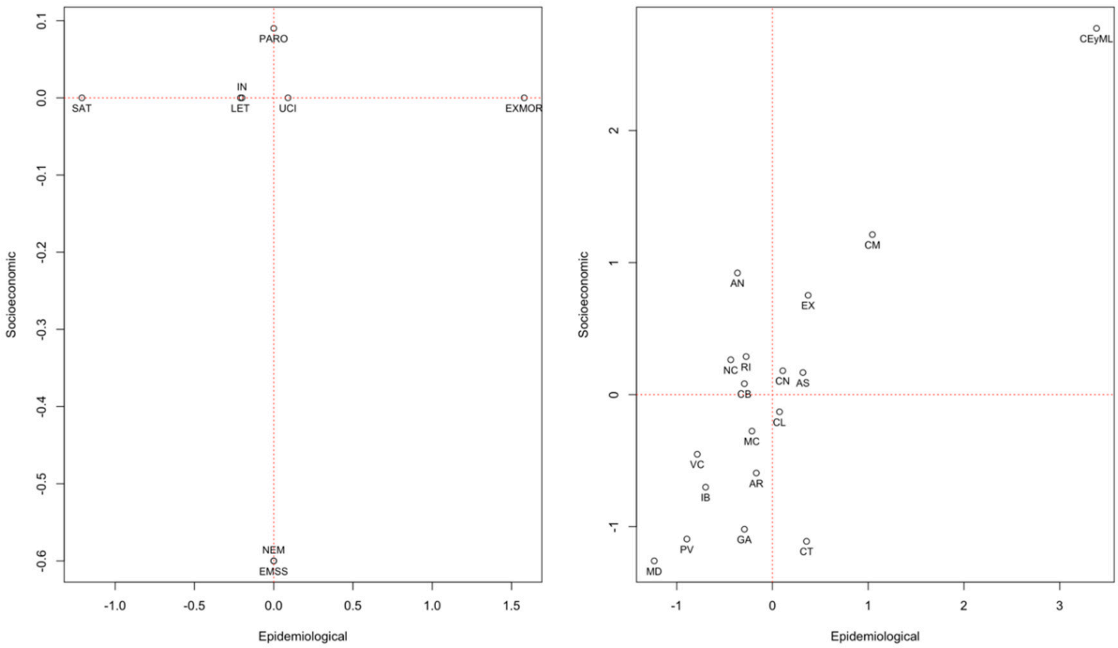

Socio economic Indicators
PARO: unemployment rate; EMSS: percentage change in companies registered with Social Security; NEM: percentage change in new companies created.
Epidemiological indicators
IN: incidence; SAT: bed saturation; LET: lethality; ICU: rate of COVID-19 admissions among COVID-19 cases; EXMOR: excess mortality.

AN: Andalusia; AR: Aragon; AS: Asturias; IB: Balearic Islands; CN: Canary Islands; CB: Cantabria; CM: Castilla La-Mancha; CL: Castilla y León; CT: Catalonia; CEyML: Ceuta and Melilla; VC: Community of Valencia; EX: Extremadura; GA: Galicia; RI: La Rioja; MD: Community of Madrid; MC: Murcia; NC: Navarra; PV: Basque Country

Period Q2-2020

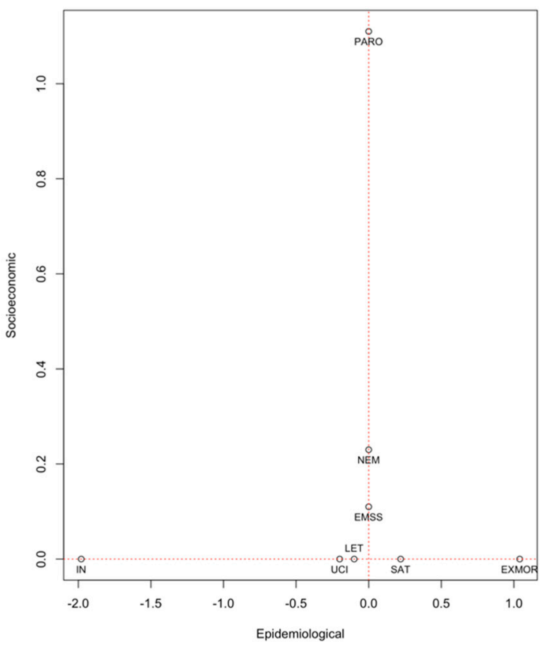

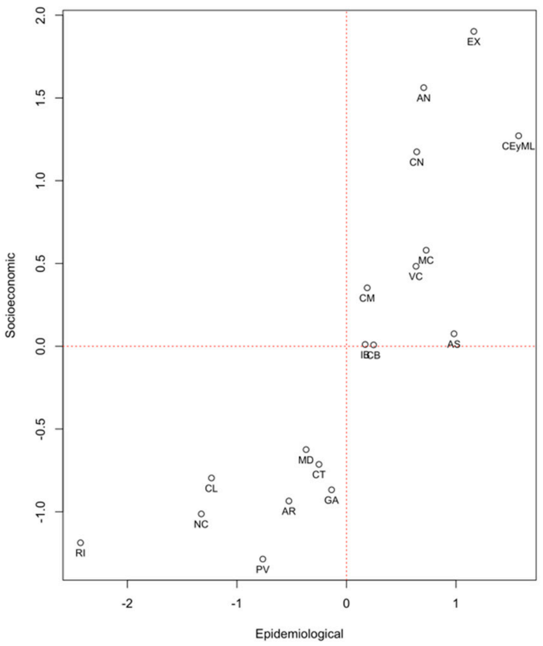

Socio economic Indicators
PARO: unemployment rate; EMSS: percentage change in companies registered with Social Security; NEM: percentage change in new companies created.
Epidemiological indicators
IN: incidence; SAT: bed saturation; LET: lethality; ICU: rate of COVID-19 admissions among COVID-19 cases; EXMOR: excess mortality.

AN: Andalusia; AR: Aragon; AS: Asturias; IB: Balearic Islands; CN: Canary Islands; CB: Cantabria; CM: Castilla La-Mancha; CL: Castilla y León; CT: Catalonia; CEyML: Ceuta and Melilla; VC: Community of Valencia; EX: Extremadura; GA: Galicia; RI: La Rioja; MD: Community of Madrid; MC: Murcia; NC: Navarra; PV: Basque Country

Period Q3-2020

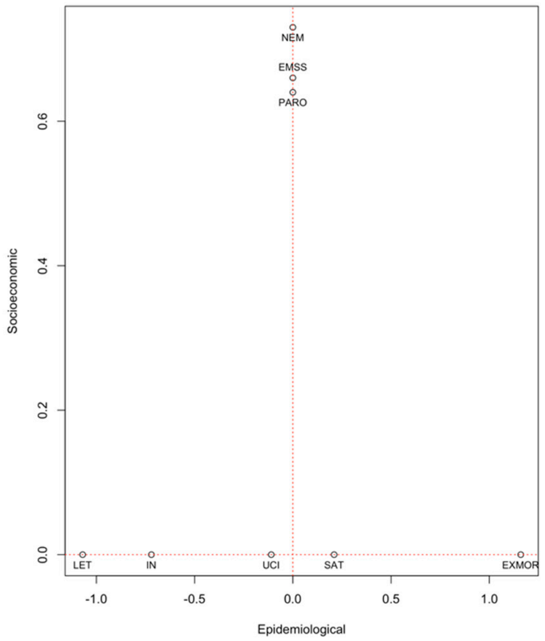

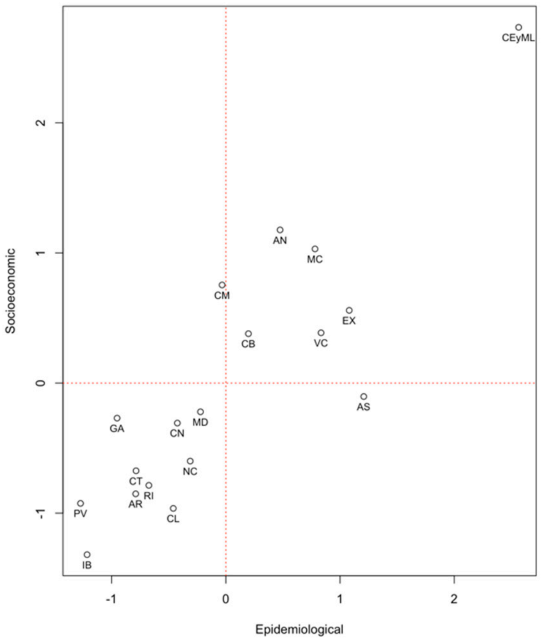

Socio economic Indicators
PARO: unemployment rate; EMSS: percentage change in companies registered with Social Security; NEM: percentage change in new companies created.
Epidemiological indicators
IN: incidence; SAT: bed saturation; LET: lethality; ICU: rate of COVID-19 admissions among COVID-19 cases; EXMOR: excess mortality.

AN: Andalusia; AR: Aragon; AS: Asturias; IB: Balearic Islands; CN: Canary Islands; CB: Cantabria; CM: Castilla La-Mancha; CL: Castilla y León; CT: Catalonia; CEyML: Ceuta and Melilla; VC: Community of Valencia; EX: Extremadura; GA: Galicia; RI: La Rioja; MD: Community of Madrid; MC: Murcia; NC: Navarra; PV: Basque Country

Period Q4-2020

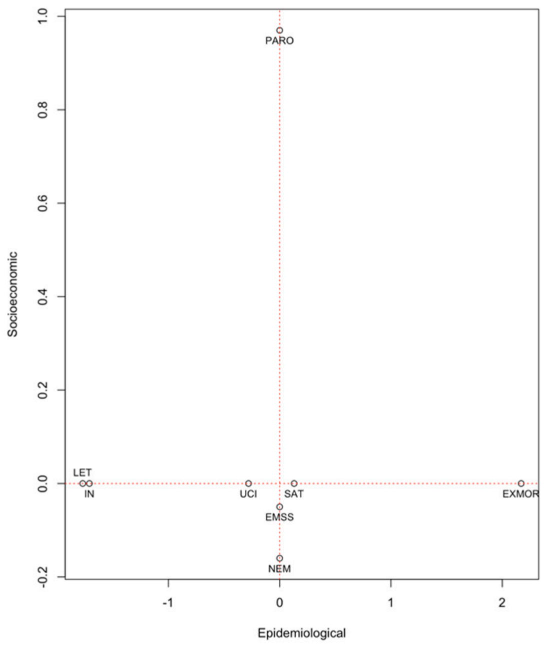

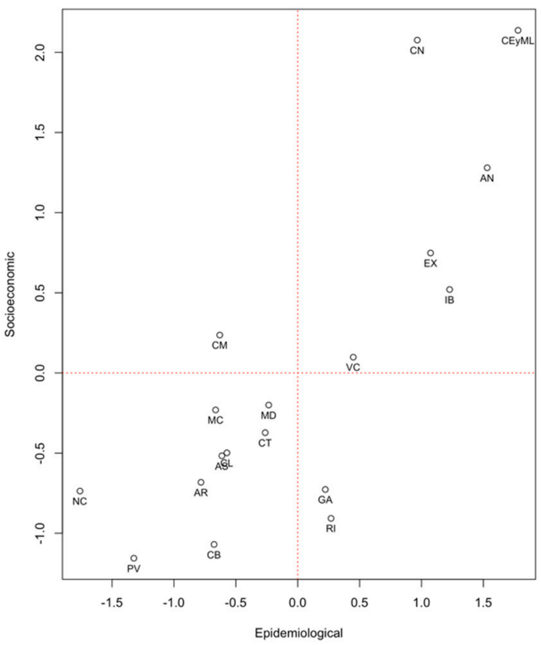

Socio economic Indicators
PARO: unemployment rate; EMSS: percentage change in companies registered with Social Security; NEM: percentage change in new companies created.
Epidemiological indicators
IN: incidence; SAT: bed saturation; LET: lethality; ICU: rate of COVID-19 admissions among COVID-19 cases; EXMOR: excess mortality.

AN: Andalusia; AR: Aragon; AS: Asturias; IB: Balearic Islands; CN: Canary Islands; CB: Cantabria; CM: Castilla La-Mancha; CL: Castilla y León; CT: Catalonia; CEyML: Ceuta and Melilla; VC: Community of Valencia; EX: Extremadura; GA: Galicia; RI: La Rioja; MD: Community of Madrid; MC: Murcia; NC: Navarra; PV: Basque Country

Period Q1-2021

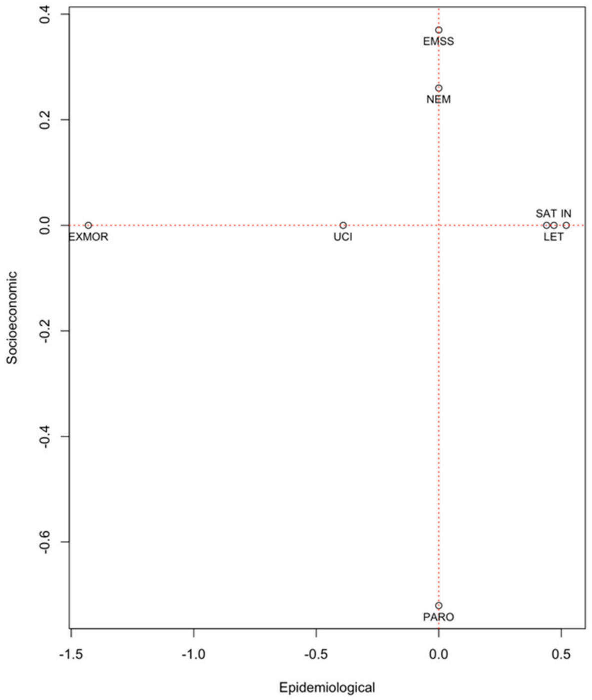

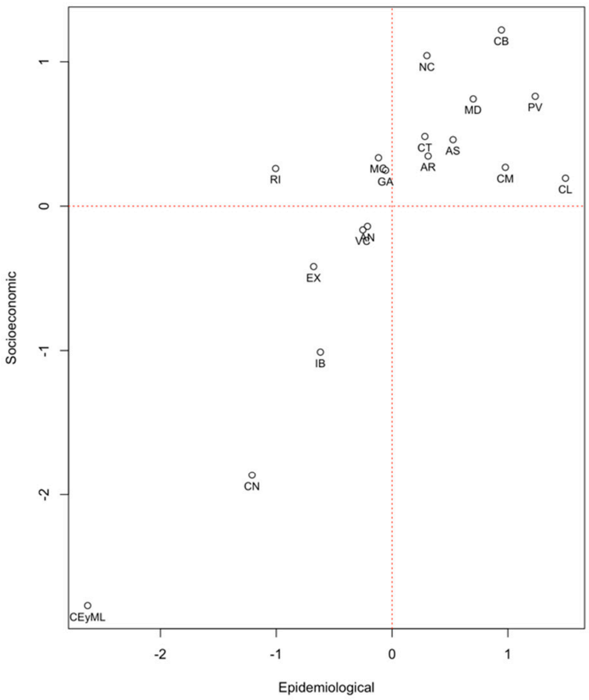

Socio economic Indicators
PARO: unemployment rate; EMSS: percentage change in companies registered with Social Security; NEM: percentage change in new companies created.
Epidemiological indicators
IN: incidence; SAT: bed saturation; LET: lethality; ICU: rate of COVID-19 admissions among COVID-19 cases; EXMOR: excess mortality.

AN: Andalusia; AR: Aragon; AS: Asturias; IB: Balearic Islands; CN: Canary Islands; CB: Cantabria; CM: Castilla La-Mancha; CL: Castilla y León; CT: Catalonia; CEyML: Ceuta and Melilla; VC: Community of Valencia; EX: Extremadura; GA: Galicia; RI: La Rioja; MD: Community of Madrid; MC: Murcia; NC: Navarra; PV: Basque Country
